# Children and adults can suspend core principles about objects and agents given a small amount of counterevidence on screen

**DOI:** 10.1038/s41598-025-31534-9

**Published:** 2025-12-13

**Authors:** Rongzhi Liu, Fei Xu

**Affiliations:** 1 Department of Psychology, University of Chicago, Chicago, United States; 2 Department of Psychology, University of California, Berkeley, United States

**Keywords:** Core knowledge, Intuitive physics, Intuitive psychology, Statistical learning, Suspending core principles, Psychology, Human behaviour

## Abstract

Human children and adults learn from statistical evidence and acquire new knowledge across many domains. Under some circumstances, human adults and nonhuman animals can suspend perceptual and cognitive priors given counterevidence. Can human learners also suspend core principles that guide our reasoning about objects and agents starting in infancy (e.g., objects are solid and cannot pass through each other; agents take the most efficient path to accomplish their goal)? In 12 experiments, we found that adults (N = 209 for physical principles; N = 189 for psychological principles) and 4- to 6-year-old children (N = 96 for physical principles; N = 96 for psychological principles) suspended these core principles on a screen when provided with as few as 3 to 6 pieces of counterevidence of each principle. Participants more readily accepted the counterevidence and were more likely to generalize counterintuitive principles for psychological than physical principles. These findings are consistent with two different conclusions: The first challenges the core knowledge view and demonstrates the power and flexibility of human learning, and the second suggests humans apply sophisticated reasoning to on-screen events. This study paves the way for future studies to test whether humans can suspend core principles in real-world contexts.

**Supplementary Information:**

The online version contains supplementary material available at 10.1038/s41598-025-31534-9.

## Introduction

Human children and adults are remarkable learners. A rich body of research shows that throughout development, we use new evidence to acquire new knowledge in many domains such as causal learning, physical and psychological reasoning, language, and mathematical cognition^1–11^.

Cognitive developmental theories have suggested that some of our knowledge, our perceptual and cognitive primitives, are innate and deeply entrenched (i.e., they are present early in infancy and shared with some nonhuman animals), and they provide the foundation for later learning^12–18^. Are these perceptual and cognitive primitives robust against counterevidence, or can they be suspended (or even modified) given new experiences and new evidence? Past research suggests that human adults can suspend perceptual priors. After being given haptic information that suggests a new light source 30 degrees away from their ‘light-from-above’ prior, they adapted quickly when asked about the light source. Furthermore, adults did not simply learn a context-specific response, as they generalized the new light source to a different task^19^. Thus, human learners can suspend the ‘light-from-above’ perceptual prior given counterevidence, and learn a new prior and generalize it to a new environment.

If human learners can suspend innate perceptual priors, can we also suspend our deeply entrenched, innate cognitive primitives given counterevidence? These cognitive primitives, often referred to as ‘core knowledge’^13,16–18^, can be observed in human infants^2,18^, are universal across cultures^20,21^, and are also shared with some nonhuman animals^14,22^. Two core knowledge systems are central to our understanding of the world, namely physical and psychological principles that guide our reasoning about objects and agents (people), respectively. Across many studies, infants have been found to respond to events inconsistent with these principles. For instance, infants find it unexpected when a toy car appears to have passed through a solid wall (inconsistent with the principle of solidity)^16^ or when a block appears to have launched another block without coming into contact with it (inconsistent with the contact principle)^23^. Infants also find it unexpected when an agent changes their goal and reaches for an object different from what prior evidence suggests (inconsistent with the goal principle)^24^ or when an agent takes an inefficient path to reach their goal (inconsistent with the efficiency principle)^25^. These core knowledge principles are shared with some nonhuman animals^14,22^, but past research provides mixed evidence on whether they can be suspended or revised^26,27^.

What about human learners? A past study found that adults perceived object-launching events as causal even when the objects did not make contact, suggesting that adults can suspend the contact principle^28^. However, no prior research has systematically examined whether observing multiple pieces of counterevidence to the core physical and psychological principles can lead human learners to suspend these principles and learn new, counterintuitive ones. Here we present 12 experiments that investigated whether children and adults can suspend these core principles given counterevidence on *screens*. Infants are exposed to screens from as early as 6 months of age^29^. Past research reports mixed findings on whether infants and children can learn from screens and transfer that learning to the real world. Some studies have found that toddlers and young children imitate models in videos less proficiently than those in live demonstrations^30^. They also have difficulty transferring information about the location of a hidden toy from videos to the real world, a phenomenon known as the video deficit effect^31,32^. However, other studies suggest that children can successfully transfer this information if they engage in contingent interaction with the videos^33,34^. Furthermore, 4- to 6-year-olds can transfer the solution of a physics problem they learned from virtual objects on a touchscreen to their interaction with real 3D objects^35^. We revisit this issue in the Discussion.

Admittedly, children and adults have observed more counterevidence to the core principles on screens (e.g., in movies and video games), and they understand that video stimuli can be illusions, making it easier for them to suspend these principles on screen than in the real world. Nevertheless, evidence that human learners can suspend the core principles on screens would lay important groundwork for future studies to test whether they can also suspend them in the real world. Specifically, examining the following questions on screens would provide important insights into designing appropriate real-world stimuli in future studies. First, how much counterevidence is needed to suspend these principles on screens? The amount of counterevidence required has important implications for how entrenched the core principles are and for how much counterevidence might be needed in the real world (i.e., more than on screens). Second, how do learners interpret the counterevidence? Do they accept them at face value, believing that objects and agents do not need to obey these principles, or do they generate alternative explanations, such as hidden mechanisms , to reconcile the counterevidence? Third, are suspensions of these principles context-specific, or do learners generalize them to new situations? Fourth, are core physical and psychological principles equally likely to be suspended? Intuitively, it seems easier to accept counterevidence to psychological principles, which we can attribute to individuals’ idiosyncratic goals or preferences, compared to counterevidence to physical principles (e.g., teleporting), which we find impossible. Relatedly, the physical principles might also differ in how likely they are to be suspended. For example, the contact principle might be more readily suspended because human learners have already encountered more counterexamples in the real world (e.g., magnets, remote controls). These differences across domains and principles will still be relevant in the real world and have important implications for how we characterize core knowledge systems. Lastly, are there developmental differences? Since adults have encountered more evidence for these principles than children, and perhaps also more counterexamples, both on screen and in the real world, does that make them more or less likely to suspend them?

In our experiments, we first measured children’s and adults’ prior beliefs about objects and agents (i.e., their default expectations) by asking a group of participants who did not observe any new evidence to make predictions about how objects and agents should behave on screens. We predict that they would expect objects and agents to obey core physical and psychological principles^3,7,8,23,25,36–43^. Then we investigated our key research question: Would children and adults suspend their default expectations about core physical and psychological principles on screens, given a small amount of counterevidence? We showed adults and 4- to 6-year-old children a few events inconsistent with the core physical principles (solidity, continuity, and contact) or the core psychological principles (goal, efficiency, and sampling), then we asked them to make predictions about new scenarios involving objects and agents. We also examined how they reasoned about the counterevidence to the physical and psychological principles by asking them to explain the events, a common method to probe children’s and adults’ reasoning^44–48^.

### Adults and children can suspend core principles about objects

**Fig. 1:**
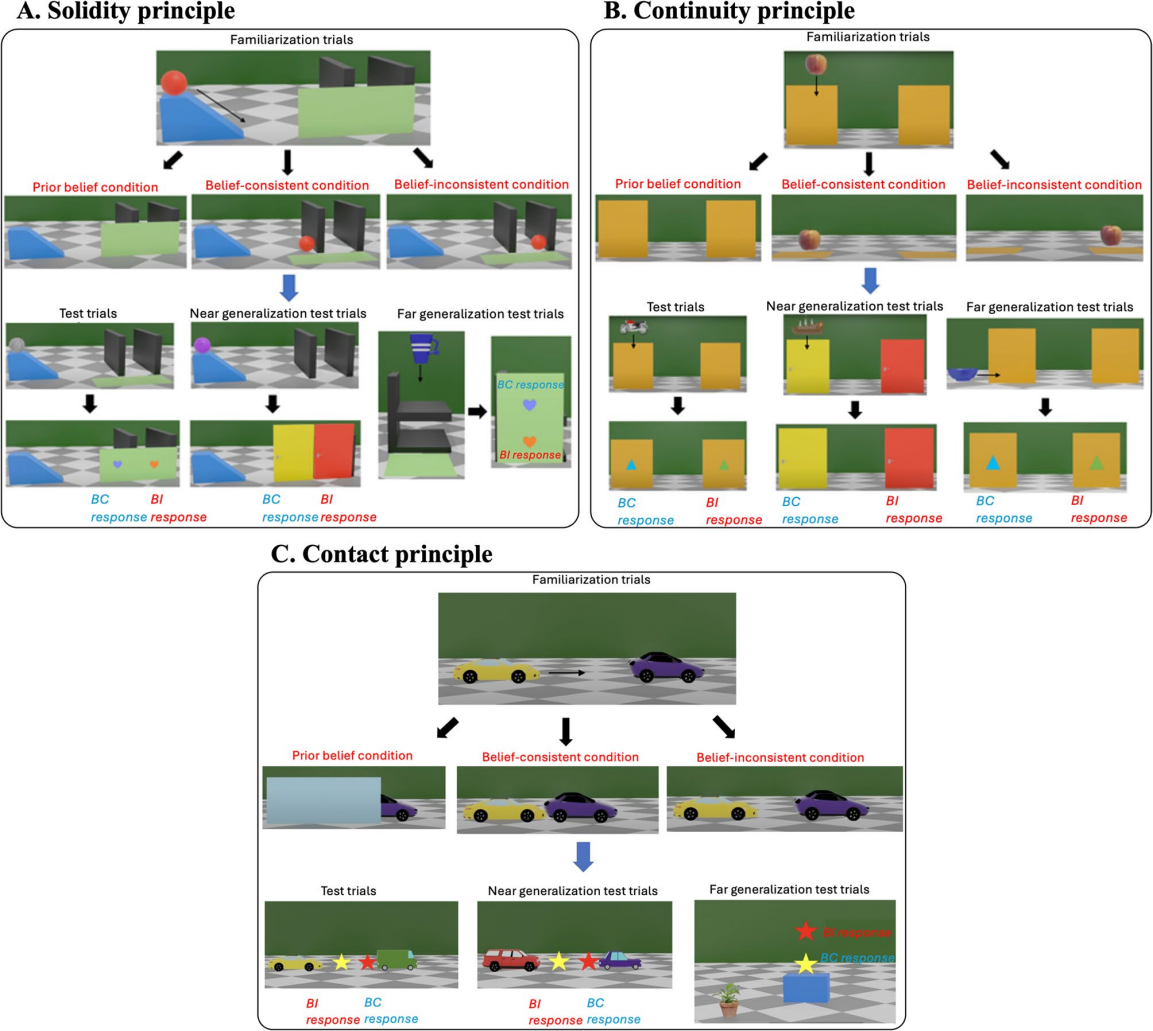
The familiarization trials and the test trials for the physical principles. The stimuli shown were used in Experiments 3 and 6. The stimuli used in Experiments 1, 2, 4, and 5 were similar and 2-dimensional. Stimuli videos were modeled after many previous studies in early development, and are available at https://osf.io/f4wmn/overview?view_only=38afbc85d0a34e25ab86cfb28e4edbe8. (**A**) Solidity principle: In familiarization trials, participants saw an object rolled down a ramp and went behind a screen with two vertical walls. When the screen was lifted, the object either appeared to have been stopped by the first wall (Belief-consistent outcome) or to have passed through the first wall (Belief-inconsistent outcome). Then participants predicted the location of new objects in similar events. In test trials, an object moved behind the same screen. In near generalization test trials, an object moved behind two doors occluding the vertical walls. In far generalization test trials, an object moved behind a screen occluding two horizontal walls. **(B)** Continuity principle: In familiarization trials, participants saw two screens and an object was lowered behind one of them. When the screens were lifted, the object was either behind the same screen (Belief-consistent outcome) or the other screen (Belief-inconsistent outcome). Then participants predicted the location of new objects in similar events. In test trials, an object was lowered behind one of the screens. In near generalization test trials, an object was lowered behind one of two doors. In far generalization test trials, an object moved horizontally behind one of the screens. **(C)** Contact principle: In familiarization trials, participants saw two objects, one moving towards the other. The second object started to move either after it had been contacted by the first object (Belief-consistent outcome) or before it had been contacted by the first object (Belief-inconsistent outcome). Then participants predicted where the first object should stop to launch/activate the second object in similar events. In test trials, the same object was launching a new object. In near generalization test trials, a new object was launching another new object. In far generalization test trials, a new object was activating a music toy.

In Experiments 1 to 3 with adults and Experiments 4 to 6 with 4- to 6-year-old children, we examined whether learners could suspend three core principles about objects: solidity (solid objects cannot pass through each other)^16^; continuity (objects traverse spatiotemporally connected paths)^49^; and contact (objects cannot interact at a distance)^23^. On a computer screen, participants observed events consistent with the principles in the belief-consistent (BC) condition, events inconsistent with the principles in the belief-inconsistent (BI) condition, or events without the critical outcomes in the prior belief (PB) condition (a between-subjects design). Then, they made binary predictions about new events that differed progressively more from the familiarization trials—in test trials (events with the same object or occluder), near generalization test trials (events with a different object or occluder), and far generalization test trials (a different type of events) (Fig. 1). In addition, participants in the BC and the BI conditions were asked to provide explanations for a familiarization event, and participants in the PB condition were asked to provide explanations for their predictions on a test trial.

**Fig. 2:**
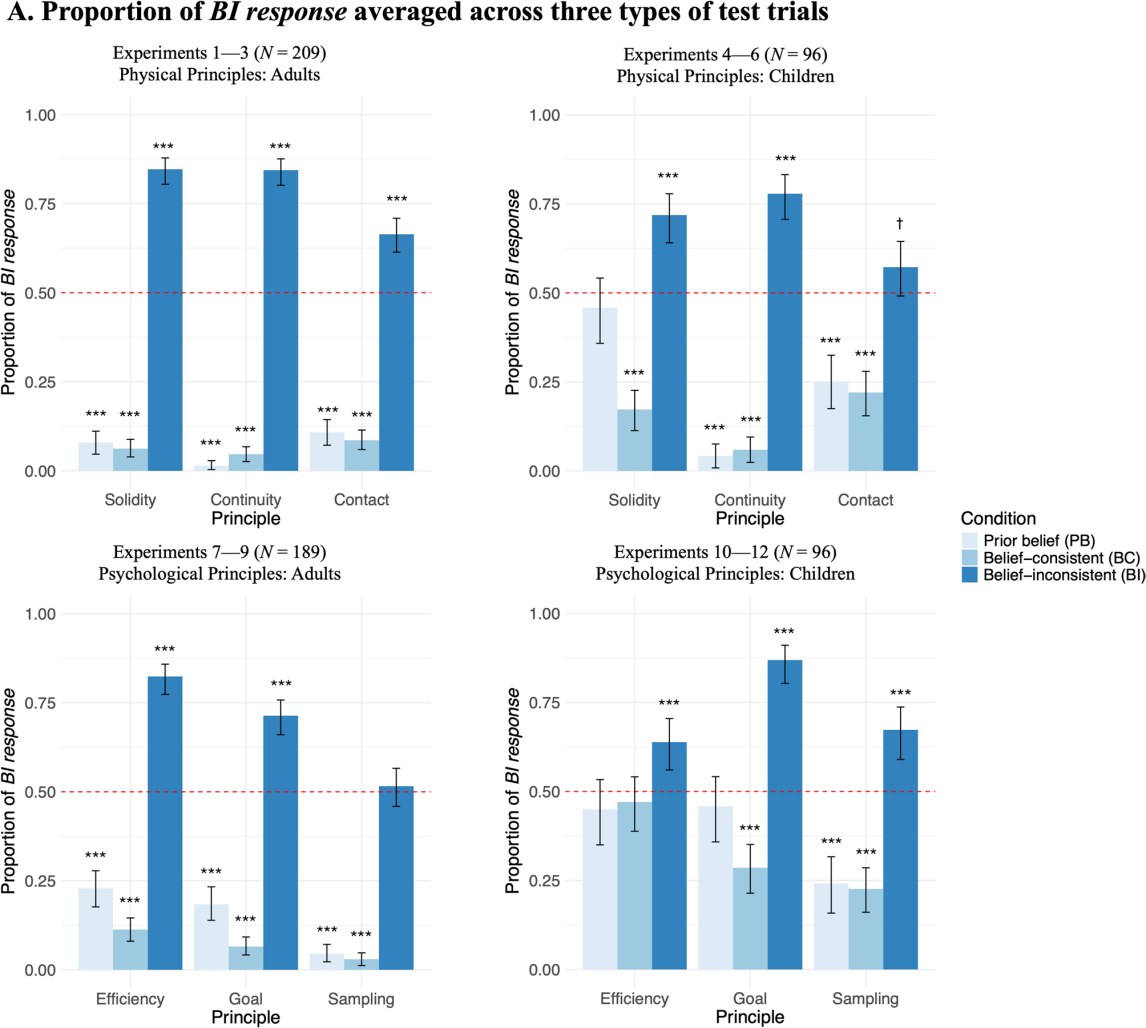

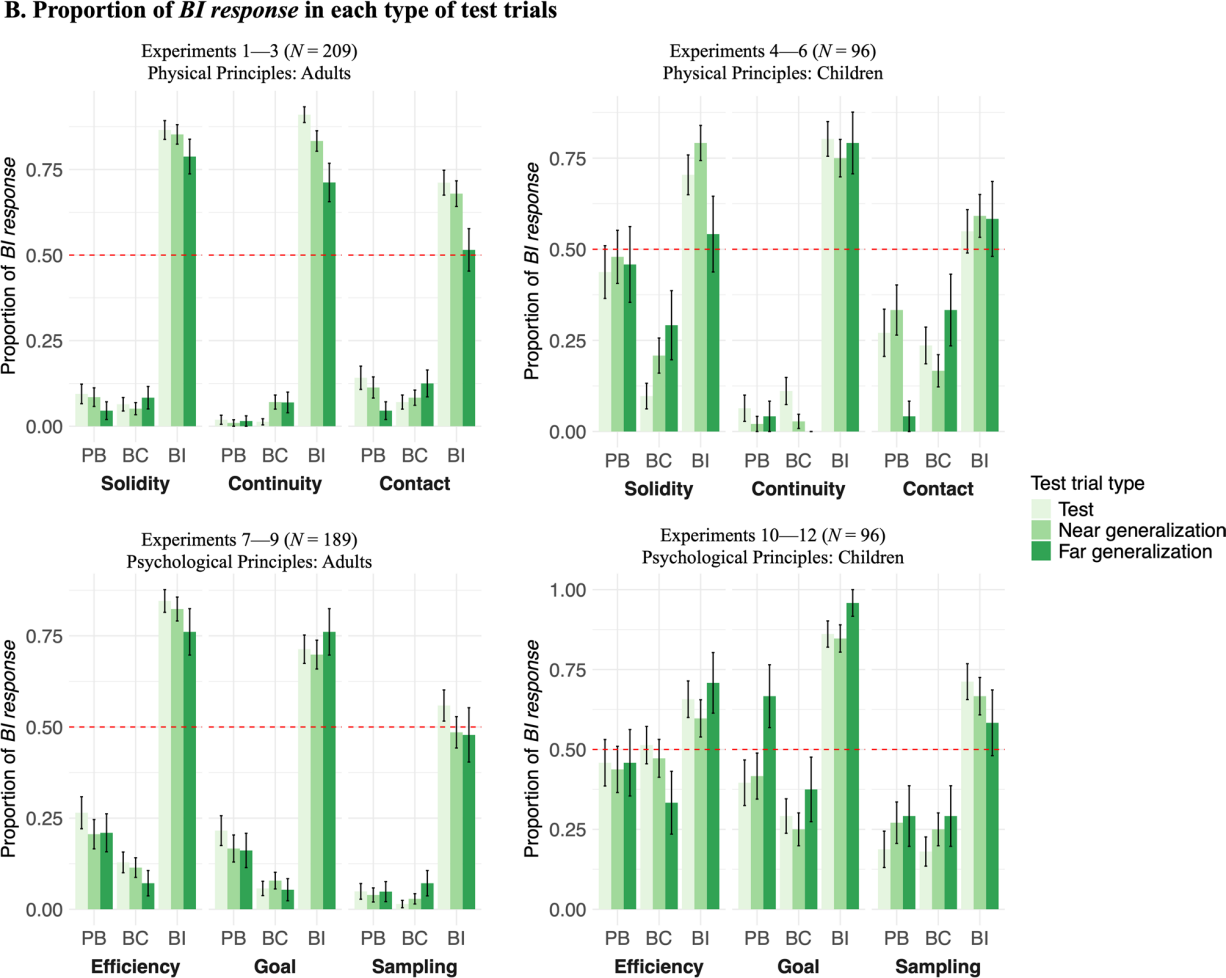
Adults’ and children’s choices in test trials for physical and psychological principles. Bar plots showing the proportion of trials that participants selected the *belief-inconsistent (BI) response* **(A)** averaged across three types of test trials and **(B)** separately for each type of test trials. Bars are grouped by condition (prior belief [PB], belief consistent [BC], belief inconsistent [BI]), principle, and experiment. The dashed line indicates chance performance (0.5), and the error bars indicate bootstrapped 95% CIs. Exact binomial tests compared participants’ average responses against chance: *** *p* < .001, † p < .08.

As shown in Fig. 2, we found that adults expected objects to follow core principles when they observed no evidence or belief-consistent evidence, but expected them to follow counterintuitive principles after observing belief-inconsistent evidence. Children’s results were similar but noisier.

We used mixed-effect logistic regression to examine the fixed effects of condition, principle, test trial type, amount of evidence, age group, and their interactions on participants’ choices, with participants’ ID as random effects. Most importantly, we found a main effect of condition: For all 3 principles, across all 3 types of test trials, adults and children were more likely to choose the *BI response* in the BI condition than in the PB condition (*β*_*continuity*_ = 8.08, *β*_*solidity*_ = 6.14, *β*_*contact*_ = 4.30, *p*s < .001) and the BC condition (*β*_*continuity*_ = 7.36, *β*_*solidity*_ = 6.87, *β*_*contact*_ = 5.10, *p*s < .001). Their choices did not differ between the PB and the BC conditions (*p*s > .05). There was also an interaction of condition and test trial type: In the BI condition, they were less likely to choose the *BI response* in far generalization test trials than in test trials (*β* = -0.95, *p* < .001) and near generalization test trials (*β* = -0.78, *p* < .001).

We also found a three-way interaction of age, condition, and principle: In the PB and BC conditions, children were more likely than adults to choose the *BI response* for Solidity (*β*_*PB*_ = 3.16, *p* < .001; *β*_*BC*_ = 1.64, *p* = .001) and Contact (*β*_*PB*_ = 1.57, *p* = .005; *β*_*BC*_ = 1.69, *p* < .001) principles. In the BI condition, children were less likely than adults to choose the *BI response* for Continuity (*β* = -1.01, *p* = .024) and Solidity (*β* = -1.32, *p* = .003) principles. No other age effects were found. There were no effects of the amount of evidence (4 vs. 6 familiarization trials) on adults’ or children’s choices (*p*s > .05).

**Fig. 3:**
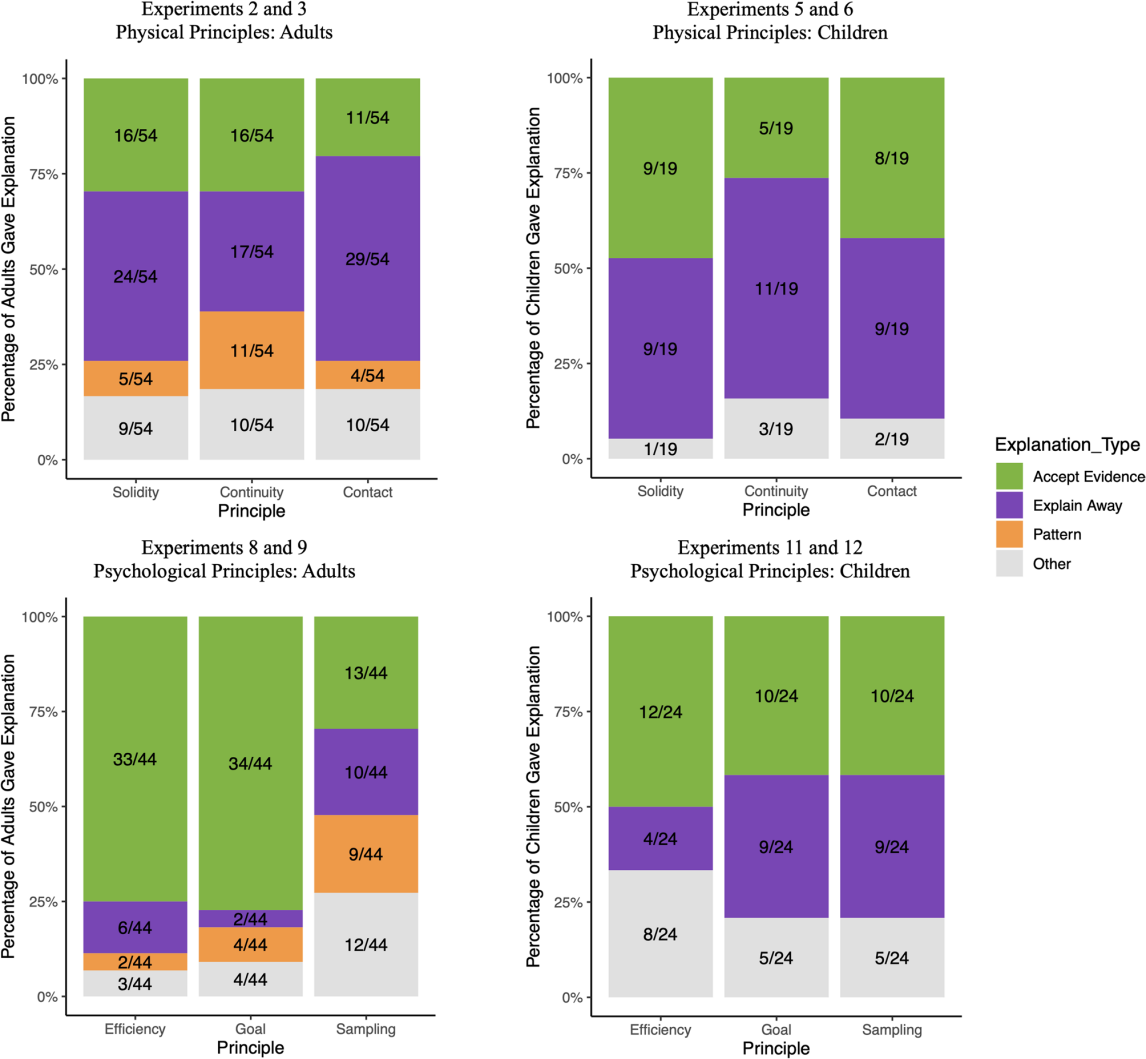
Adults and children’s explanations for physical and psychological principles in the belief-inconsistent (BI) condition. Stacked bar plots showing the percentage of explanations participants provided by explanation type and principle, in Experiments 2–3, Experiments 5–6, Experiments 8–9, and Experiments 11–12.

Two researchers coded participants’ explanations into different categories (Cohen’s Kappa = 0.94 for adults, 0.93 for children). In the PB and BC conditions, a majority of adults referred to the principle itself to explain the evidence (PB: 98.9%; BC: 98.2%); one adult also mentioned “Because that is how objects work in the real-life”. Most children also referred to the principle (PB: 58.3%; BC: 69.8%), and the other responses were irrelevant to the principle or incomprehensible (Table 1). In the BI condition, responses were coded into 4 categories based on the criteria in Table 2. Within the *explain away* category, a few adults mentioned that they thought the videos had been edited. The distribution of explanation types for adults and children did not differ from each other (*p* > .10) (Fig. 3). For adults, those who provided *accept evidence* and *pattern* explanations were more likely to choose the *BI response* for the principle compared to participants who provided *explain away* explanations (*β*_*accept evidence*_ = 0.98, *p* = .03; *β*_*pattern*_ = 1.52, *p* = .02) or *other* explanations (*β*_*accept evidence*_ = 0.96, *p* = .04; *β*_*pattern*_ = 1.50, *p* = .03) . For children, with fewer explanations provided, the type of explanation did not significantly predict their choices in the test trials. See the Supplemental Materials for detailed results.

**Table 1:** Coding criteria and examples for explanations in the prior belief (PB) and belief-consistent (BC) conditions.

	Criterion		Examples
Principle	Referred to the corresponding principle to explain the event	Adults	Solidity: The ball couldn’t pass through the solid wall and stopped in front of that wall and remained in that location Continuity: Because that is where it fell and we didn’t see it move towards the other square Contact: The yellow car needs to touch the green car in order to make it move Efficiency: The red ball wants to get to yellow in the shortest way possible so without the obstacle it just goes directly there Goal: The pink kid probably likes the trumpet no matter where it is placed Sampling: The green kid is going out of their way to attain the yellow toys from the pink ones which shows a preference for the yellow toy
		Children	Solidity: Because it’s kind of like it gets blocked by the grey one Continuity: Because I saw it go there Contact: Because it pushes it and then it goes, goes, goes Efficiency: Because it’s easier Goal: Because he likes the fox better Sampling: Because he grabbed 4 pink ones and 0 yellow ones
Other	Explanations irrelevant to the principle or incomprehensible	Adults	Solidity: Because it rolled with speed Continuity: Not sure Contact: Because of the speed Efficiency: Because it jumped Goal: Guessed Sampling: Boring
		Children	Solidity: I don’t know Continuity: Maybe it is going to hide from a monster Contact: Red means stop Efficiency: Because he’s red Goal: Because I love it Sampling: Because it’s the silly one

**Table 2:** Coding criteria and examples for each type of explanations in the belief-inconsistent (BI) condition.

	Criterion		Examples
Accept Evidence	Accepted the counterevidence to the target principle	Adults	Solidity: The ball went through the first wall Continuity: The apple jumped to the right screen Contact: The yellow car goes behind the purple car without touching it and it launches off Efficiency: The kid continues to take the jump because he enjoys it more Goal: The kid only wanted toys from the left side Sampling: The kid likes to weed out the toys he does not like from the big box
		Children	Solidity: The ball went through the wall Continuity: Because it went from the left side to the right side Contact: Because it stopped there and moved the purple car Efficiency: Because it was fun to jump Goal: Because it liked that side more than that side Sampling: Because he likes it better
Explain Away	Explained the counterevidence with reasons that would not involve any violations of the target principle	Adults	Solidity: The first wall was farther behind the screen, leaving a gap for the ball to pass through Continuity: There is an underground passage Contact: The purple car got scared of the yellow car potentially hitting it Efficiency: The kid thought there was still a wall Goal: Because the bear moved places and the cartoon could only see one toy based on how his eyes were drawn Sampling: Got bored of the other toy
		Children	Solidity: There is a hole in the first wall Continuity: Maybe the screens are magic Contact: Because the purple car didn’t want the yellow car to bump into it Efficiency: There is an invisible wall in the middle Goal: Because he was done playing with the soccer ball Sampling: Because he wants to play with another type of toy
Pattern	Noted the pattern in the evidence	Adults	Solidity: Always appeared behind second wall from where it started Continuity: That has been the pattern the whole time; for the object to show up at the opposite door Contact: It has been the pattern the whole time for that to happen Efficiency: Because that is what the red kid had previously done Goal: Three times of one toy, the fourth time picks the other toy Sampling: Every fifth toy they pick the opposite
		Children	None
Other	Explanations that cannot be categorized into the first three categories	Adults	Solidity: The way it goes Continuity: I am not sure Contact: Physics? Efficiency: When there is no wall the parabola is smaller Goal: I have no idea Sampling: Not as many
		Children	Solidity: I don’t know Continuity: I don’t know Contact: Because there is a red light and the car can’t go Efficiency: Because the path matched his color Goal: Maybe he just wanted to go outside and play with his friends and his favorite sport was soccer Sampling: I don’t know

In Experiments 1–6, we found that adults and children expected objects to obey the core physical principles on screen. When given a small amount of counterevidence, both adults and children suspended these principles and learned new, counterintuitive principles about objects on the screen. They also generalized counterintuitive principles to new situations, but were more conservative when applying them to situations that differed more from the original counterevidence. Adults who accepted the counterevidence were also more likely to learn counterintuitive principles.

### Adults and children can suspend core principles about agents

**Fig. 4:**
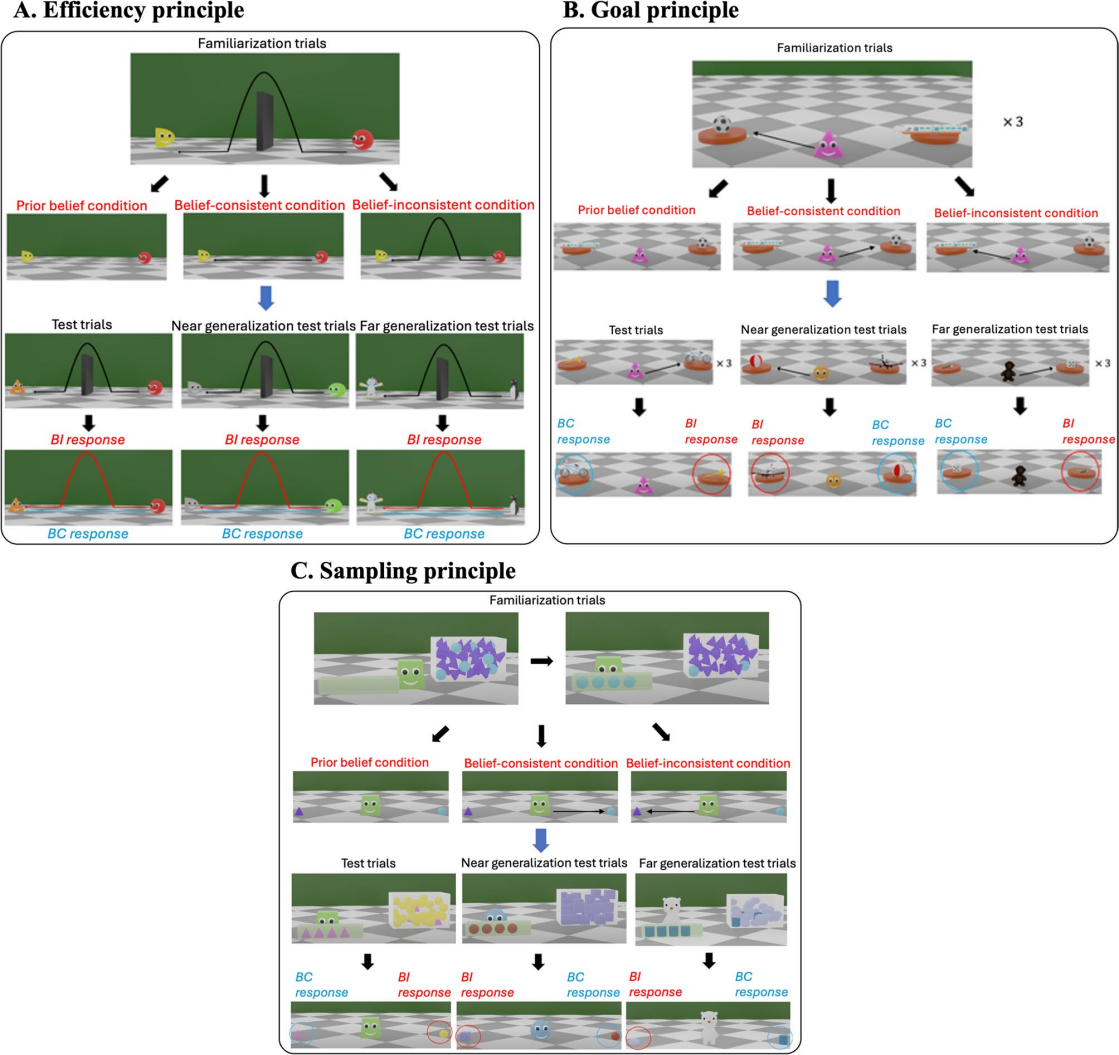
The familiarization trials and the test trials for the psychological principles. The stimuli shown were used in Experiments 9 and 12. The stimuli used in Experiments 7, 8, 10, and 11 were similar and 2-dimensional. Stimuli videos were modeled after many previous studies in early development, and are available at https://osf.io/f4wmn/overview?view_only=38afbc85d0a34e25ab86cfb28e4edbe8. (**A**) Efficiency principle: In familiarization trials, participants saw an event where two agents were separated by a wall. One agent moved towards the other agent by jumping over the wall. Then the wall was removed. The agent either moved toward the other agent by taking the most efficient path, i.e., in a straight line (belief-consistent outcome), or by taking an inefficient path, i.e., jumping as if the wall had been present (belief-inconsistent outcome). In test trials, participants were shown similar events and made predictions about which path agents would take to move toward their goals. For all 3 principles, participants made predictions about the same agent in test trials, new geometric-shaped agents in near generalization test trials, and new animal agents in far generalization test trials. **(B)** Goal principle: In familiarization trials, participants saw an event in which an agent repeatedly chose one of two objects. Then the two objects switched positions. The agent chose either the same object (belief-consistent outcome) or the other object (belief-inconsistent outcome). Then participants were shown similar events and made predictions about which objects the agents would choose. **(C)** Sampling principle: In familiarization trials, participants saw an event in which an agent picked out four objects that were clearly the minority type in the toy box. Then when given a choice between the minority object and an object that was in the majority in the toy box, the agent chose either the minority object (belief-consistent outcome) or the majority object (belief-inconsistent outcome). Then participants were shown similar events and made predictions about which object the agents would prefer.

In Experiments 7 to 9 with adults and Experiments 10 to 12 with 4- to 6-year-old children, we examined whether learners could suspend three core principles about agents: efficiency (agents take the most efficient path to accomplish their goal)^25^; goal (agents’ actions are goal-directed)^24^; and sampling (when an agent chooses objects that are in the minority of a population, they prefer that type of object)^50^. The design and procedure were the same as Experiments 1–6. Participants made predictions about agents that differed progressively more from the agents in the familiarization trials—in test trials (the same agent), near generalization test trials (new geometric-shaped agents), and far generalization test trials (new animal agents) (Fig. 4).

As shown in Fig. 2, we found that adults expected agents to follow core principles when they observed no evidence or belief-consistent evidence, but expected them to follow counterintuitive principles after observing belief-inconsistent evidence (except for the Sampling principle). Children’s results were similar but noisier.

Mixed-effect logistic regression with the same fixed and random effects as in Experiments 1–6 revealed an interaction of condition and age group: for all 3 principles, across all 3 types of test trials, both adults and children were more likely to choose the *BI response* in the BI condition than in the PB condition (adults: *β*_*efficiency*_ = 4.18, *β*_*goal*_ = 3.68, *β*_*sampling*_ = 4.36, *p*s < .001; children: *β*_*efficiency*_ = 1.25, *p* = .019; *β*_*goal*_ = 2.80, *p* < .001; *β*_*sampling*_ = 2.66, *p* < .001) and the BC condition (adults: *β*_*efficiency*_ = 5.27, *β*_*goal*_ = 5.30, *β*_*sampling*_ = 5.03, *p*s < .001; children: *β*_*efficiency*_ = 0.96, *p* = .040; *β*_*goal*_ = 3.63, *p* < .001; *β*_*sampling*_ = 2.65, *p* < .001). Adults were less likely to choose the *BI response* in the BC condition than in the PB condition (*β*_*efficiency*_ = -1.21, *p* = .006; *β*_*goal*_ = -1.61, *p* < .001; not significant for Sampling: *β*_*sampling*_ = -0.71, *p* = .24), and children’s responses did not differ between the PB and the BC conditions (Efficiency: *β*_*efficiency*_ = 0.31, *β*_*goal*_ = -0.85, *β*_*sampling*_ = -0.001, *p*s > .10). Unlike the physical principles, we did not find an interaction of condition and test trial type for the psychological principles, suggesting that adults and children were equally likely to choose the *BI response* in all 3 types of test trials (Fig. 2).

There was also an interaction of condition and amount of evidence: In the BI condition, when adults were given 6 familiarization trials, they were more likely to choose the *BI response* in the test trials, compared to when they were given 3 familiarization trials (*β* = 0.83, *p* = .046). The amount of evidence did not affect children’s choices in the BI condition, or adults’ and children’s choices in the BC condition (*p*s > .05).

We also found a three-way interaction of age, condition, and principle: In the PB and BC conditions, children were more likely than adults to choose the *BI response* for all 3 principles (PB: *β*_*efficiency*_ = 1.41, *p* = .006; *β*_*goal*_ = 1.82, *p* = .005; *β*_*sampling*_ = 2.36, *p* < .001; BC: *β*_*efficiency*_ = 2.83, *β*_*goal*_ = 2.55, *β*_*sampling*_ = 3.12, *p*s < .001). In the BI condition, children were less likely than adults to choose the *BI response* for the Efficiency principle (*β* = -1.51, *p* < .001), and more likely than adults to choose the *BI response* for the Goal principle (*β* = 0.95, *p* = .037). No other age effects were found.

Three researchers coded participants’ explanations into different categories (Light’s Kappa = 0.72 for adults, 0.81 for children). In the PB and BC conditions, a majority of adults referred to the principle itself to explain the evidence (PB: 97.6%; BC: 94.4%). Most children also referred to the principle (PB: 66.7%; BC: 73.3%), and the other responses were irrelevant to the principle or incomprehensible. In the BI condition, responses were coded into 4 categories based on the criteria in Table 2. The distribution of explanation types differed for adults and children (Fig. 3). Adults were more likely to provide *accept evidence* than *explain away* explanations compared to children (*β* = 1.14, *p* = .01). Adults were more likely to choose the *BI response* if they provided *accept evidence* explanations for that principle, compared to if they provided any other types of explanations (*β*_*explain away*_ = 1.44, *p* < .001; *β*_*pattern*_ = 1.05, *p* = .017; *β*_*other*_ = 1.96, *p* < .001). Children who provided *accept evidence* explanations were more likely to choose the *BI response* for the principle compared to those who provided *other* explanations (*β* = 0.95, *p* = .01; children’s data were noisier than that of adults, see Supplemental Materials).

In Experiments 7–12, we found that adults expected agents toobey the core psychological principles on screen. In contrast, children showed weaker expectations about all 3 principles, and they only reliably expected agents to obey the sampling principle (see^51,52^ for evidence that infants and children have weaker expectations about goal and efficiency principles). Importantly, when given a small amount of counterevidence, adults suspended these principles and learned new principles about agents on the screen; children suspended the sampling principle, and were also less likely to expect agents to obey the efficiency and goal principles compared to their default expectations. Adults and children also generalized their belief-inconsistent responses to all types of test trials. Their explanation data showed that those who accepted the evidence were more likely to provide belief-inconsistent responses.

### Comparison between the physical and psychological principles: objects vs. agents

Next, we analyzed the combined results of the object and agent domains using mixed-effect logistic regression that included an additional fixed effect of domain (object vs. agent) and the interaction of domain and condition. We found that participants were more likely to choose the *BI response* for agents than for objects in the PB condition (*β* = 1.26, *p* < .001) and the BC condition (*β* = 0.89, *p* = .007), but their choices did not differ between domains in the BI condition (*β* = -0.03, *p* = .921). This suggests that adults and children had stronger prior beliefs for objects than agents, but after observing counterevidence, they were overall equally likely to make counterintuitive predictions for objects and agents.

We also compared the explanation data across the two domains. Participants were more likely to provide *accept evidence* explanations for agents than *explain away* explanations (*β* = 1.48, *p* < .001) and *pattern* explanations (marginally significant: *β* = 0.79, *p* = .052), compared to objects, and they were less likely to provide *explain away* explanations for agents than *other* explanations, compared to objects (*β* = -0.95, *p* = .005).

## Discussion

Across 12 experiments, our findings show that when given a small amount of statistical evidence that goes against core principles that even preverbal infants have about objects and agents, adults and young children can suspend these principles on screen (see also^38^, for evidence that infants think that animated agents can move discontinuously from one screen to another).

How did learners interpret the counterevidence to core physical and psychological principles in screen events? Our explanation data showed that about a third of adults and children accepted the counterevidence to the physical principles at face value, and about half of them did so for the psychological principles. These participants explicitly admitted that objects and agents can violate these principles, and they were also more likely to predict that objects and agents would follow the new, counterintuitive principles. Thus, both the verbal explanations and behavioral predictions suggest that at least a subset of learners appear to have suspended their beliefs about the core principles in our task. A second group of learners (less than half for physical principles, and less than a third for psychological principles) tried to explain away the observed counterevidence by appealing to alternative mechanisms (e.g., a hidden tunnel, an invisible wall), and were less likely to predict counterintuitive outcomes. This suggests that some learners might have thought that the violations in the videos were illusions, and were unwilling to suspend their beliefs about the principles. A small group of adults explicitly appealed to the statistical pattern in the counterevidence, suggesting that some learners might be making counterintuitive predictions based on statistical learning, but it is unclear whether they considered the core principles relevant on the screen. The explanation data should be interpreted with caution since they only capture what participants chose to say. The explanation task might also be harder for children with limited verbal ability (but it is worth noting that the pattern of responses was largely consistent between children and adults). Future research can more comprehensively probe learners’ reasoning processes when they encounter counterevidence.

We also found that learners generalized their suspensions of these principles to new contexts. For example, after watching a ball going through a wall, they expected a cup to go through a different wall; after watching a geometric-shaped agent taking an inefficient path to get to its goals, they expected an animal agent to do the same. Therefore, learners did not learn to suspend these principles just in a specific context; instead they generalized the new, counterintuitive principle to different contexts. Since we only tested limited generalization contexts on the same screen, it is still possible that learners only suspended the principles in these particular contexts. Future research can investigate whether learners are willing to generalize to more dissimilar contexts, to different screens, and even to the real world^29–35^.

Our findings revealed important differences between the core physical and psychological principles, and have theoretical implication for the characterization of the two core knowledge systems (objects and agents)^18^. Both adults and children have stronger prior beliefs for the physical principles than the psychological principles on screen. Compared with the psychological principles, learners are less willing to suspend the physical principles given the same amount of counterevidence in two ways: they are less likely to generalize the new principles to new contexts, and they are less likely to accept the counterevidence and more likely to try to explain it away. This difference is consistent with our intuition that violations of psychological principles can be attributed to individuals’ idiosyncratic goals or preferences, whereas violations of physical principles seem impossible. Therefore, even on screen, where counterevidence is generally more acceptable, learners differ in the extent to which they are willing to suspend physical vs. psychological principles. This domain difference suggests that the core knowledge systems might differ in their flexibility, and learners might be less willing to suspend and modify the object system than the agent system. This finding also implies that learners might need much more compelling counterevidence in the real world to suspend the physical principles than the psychological principles.

Our findings also revealed some interesting developmental differences. First, children had weaker prior beliefs than adults about all physical and psychological principles except continuity. Second, given the same amount of counterevidence, adults were more likely than children to suspend some of the principles (continuity, solidity, and efficiency). The simplest explanation for these differences is that children’s data were noisier than adults’ data. Children might be more likely than adults to choose randomly, and therefore their choices were closer to chance in some conditions (Fig. 2). It is also possible that these developmental differences reflect the different experiences adults and children have had with these principles. For instance, growing up in today’s world, children might have encountered a larger proportion of data violating these principles on screens (e.g., in movies and video games, and perhaps also in real life, with remote controls and the like), which might make them more uncertain about whether these principles apply on screens or not (i.e., they have a flatter, less informative prior). Therefore, children might need more evidence to form either a belief that objects and agents obey the principles, or a belief that objects and agents obey a new, counterintuitive principle. This developmental difference is also consistent with the hypothesis that it might be easier to learn violations of principles after already acquiring the principles, a possibility supported by past findings from nonhuman animals^26,27,53,54^.

What can we conclude from these findings? Our results may have important theoretical implications for the core knowledge view and the power of human learning. Given as few as 3 to 6 pieces of counterevidence, both adults and children readily suspended the core principles on screen. These findings challenge the core knowledge view, which argues that these principles are innate and deeply entrenched^17,18^. Instead, they suggest that these principles might be learned through experience^52,55^, and can be unlearned given counterevidence. Moreover, they extend the large body of literature on the power and flexibility of human learning^4,5,10,19^, and suggest that humans s can learn from counterevidence and suspend even our most entrenched beliefs.

Alternatively, rather than challenging the core knowledge view, our findings may reflect human learners’ sophisticated reasoning about events on screens^30–32^. Adults and children might believe that core physical and psychological principles do not necessarily apply to screens^56^. When given evidence for counterintuitive rules on a particular screen, they readily learned that these new rules apply to this screen, without changing their beliefs about the core principles in the real world. In addition, since we used occluders to mask actual “violation” parts of the physical events, as in many infant studies, participants might have interpreted the counterevidence as illusions or believed the videos were edited, as a few adults mentioned in their explanations. These interpretations would lead to the conclusion that human learners have a nuanced understanding of the differences between the real world and virtual screens, and they have more flexible beliefs about what can happen on screens.

An important next step is to distinguish between these two interpretations by investigating whether human learners can also suspend core principles in real-world contexts. The current work establishes critical foundations for these future studies by offering insights into how much counterevidence may be required, the types of alternative interpretations of the counterevidence, and relevant domain and developmental differences. For example, since participants often proposed hidden mechanisms (e.g., secret tunnels) to explain away counterevidence on screen, future studies with real objects could rule out these explanations by revealing all relevant parts of the stimuli before showing the counterevidence. In an ongoing study, we are testing this question with real-world stimuli adapted from past studies^57^, and our preliminary results suggest that infants can also suspend core physical and psychological principles in a real-world setting.

## Methods

### Experiments 1–3

#### Participants

In Experiment 1, we tested 47 adults (mean age = 30 years; range = 18 to 55; *SD* = 9.2; 25 females) on an online research platform, Prolific. In Experiment 2, we tested 60 adults (mean age = 33 years; range = 18 to 54; *SD* = 9.41; 35 females) on Prolific. In Experiment 3, we tested 102 undergraduate students who were enrolled in psychology courses (mean age = 20.52 years; range = 18 to 36; *SD* = 2.62; 81 females).

Experiments 1 –12 were approved by the Committee for Protection of Human Subjects of University of California, Berkeley (approval ID: 2010-03-1013). All procedures were conducted in accordance with the Helsinki declaration. Participants provided written informed consent prior to participating in the experiment. Participants were paid ($3.2 for a 20-min experiment in Experiment 1; $4 for a 25-min experiment in Experiment 2) or received course credits (Experiment 3).

#### Design

In all 3 experiments, participants were tested on 3 principles (Continuity, Solidity, and Contact) in counterbalanced orders. For each principle, participants observed a few events in the familiarization trials, and then completed a few test trials. Stimuli videos are available in the Open Science Framework (OSF) repository, https://osf.io/f4wmn/overview?view_only=38afbc85d0a34e25ab86cfb28e4edbe8. Participants in some experiments were also asked to explain the events at the end. We varied the conditions, amount of evidence, test trial types, stimuli type, and explanation questions across 3 experiments (Table 3).

**Table 3:** Design across 12 experiments.

Experiment	Principles	Total N	Age group	Conditions (N per condition)	Amount of Evidence	Test trial type	Stimuli type	Explanation questions
1	Solidity^16^ Continuity^50^ Contact^23^	47	Adults	BC (23) and BI (24)	4	TT, NGTT	2D	no
2		60	Adults	PB (20), BC (19), BI (21)	6	TT, NGTT	2D	In BC and BI conditions
3		102	Adults	PB (33), BC (36), BI (33)	6	TT, NGTT, FGTT	3D	In BC and BI conditions
4		24	Children	BC (12) and BI (12)	4	TT, NGTT	2D	no
5		36	Children	PB (12), BC (12), BI (12)	6	TT, NGTT	2D	In BC and BI conditions
6		36	Children	PB (12), BC (12), BI (12)	6	TT, NGTT, FGTT	3D	In BC and BI conditions
7	Efficiency^25^ Goal^24^ Sampling^51^	47	Adults	BC (23) and BI (24)	3	TT, NGTT	2D	no
8		60	Adults	PB (20), BC (19), BI (21)	6	TT, NGTT	2D	In BC and BI conditions
9		82	Adults	PB (31), BC (28), BI (23)	6	TT, NGTT, FGTT	3D	In BC and BI conditions
10		24	Children	BC (12) and BI (12)	3	TT, NGTT	2D	no
11		36	Children	PB (12), BC (12), BI (12)	6	TT, NGTT	2D	In BC and BI conditions
12		36	Children	PB (12), BC (12), BI (12)	6	TT, NGTT, FGTT	3D	In BC and BI conditions

For Conditions, participants observed events consistent with the principles in the belief-consistent (BC) condition, events inconsistent with the principles in the belief-inconsistent (BI) condition, or events without the critical outcomes in the prior belief (PB) condition. For test trial types, TT stands for test trials, NGTT stands for near generalization test trials, and FGTT stands for far generalization test trials.

*Conditions*. In Experiment 1, participants were randomly assigned to the Belief Consistent (BC) condition or the Belief Inconsistent (BI) condition. In Experiments 2 and 3, participants were randomly assigned to the Prior Belief (PB) condition, the BC condition, or the BI condition. In the PB condition, participants did not receive any new evidence that supported or violated the principles. In the BC condition, participants observed a few events that were consistent with each of the principles. In the BI condition, they observed a few events that violated each of the principles.

*Amount of evidence*. For each principle, participants observed 4 familiarization trials (Experiment 1) or 6 familiarization trials (Experiments 2 and 3).

*Test trial type*. There were 3 types of test trials. In test trials, participants were asked to make predictions about similar events as the ones they observed in the familiarization trials, with the same objects or the same occluders. In near generalization test trials, participants were asked to make predictions about similar events with different objects or different occluders. In far generalization test trials, participants were asked to make predictions about new events.

In Experiments 1 and 2, participants completed 2 test trials and 2 near generalization test trials, in counterbalanced orders. In Experiment 3, participants completed 2 test trials, 2 near generalization test trials, and 2 far generalization test trials, in counterbalanced orders.

In each test trial, participants chose between the *Belief Consistent (BC) response* and the *Belief Inconsistent* (*BI*) *response*. They never received feedback about whether their choices were correct or incorrect.

*Stimuli type*. In Experiments 1 and 2, we used 2 dimensional stimuli made in PowerPoint. In Experiment 3, we used 3-dimensional stimuli made in Blender.

*Explanation questions*. In Experiments 2 and 3, participants in the BC and BI conditions were asked to explain one of the familiarization events for each principle at the end of the experiment. In Experiment 3, participants in the PB condition were also asked to explain their own predictions in one of the test trials for each principle at the end of the experiment.

#### Stimuli and procedure

At the beginning of the experiment, participants were shown the following instructions: “In the following pages, you will watch some videos and observe how objects move. Then, you will be asked to make some predictions.”

#### Continuity principle

*Familiarization trials.* In each familiarization trial, two orange screens appeared side by side, with a gap in between. An object (e.g., an apple or an umbrella) appeared above one of the screens then disappeared behind it. In the PB condition, the screens were not removed to reveal the location of the object. In the other two conditions, the screens were removed. The object was either at the location of the screen that the object disappeared behind (BC condition) or at the location of the other screen (BI condition). The object was different in each trial.

*Test trials.* Participants completed the test trials next. In test trials, a new object disappeared behind one of the orange screens. A blue triangle and a green triangle appeared in front of the two screens. Participants were asked, “Where do you think you will find the [object] now? Behind the blue triangle or behind the green triangle?” Participants chose their responses, either the screen where the object disappeared behind (the *BC response*) or the other screen (the *BI response*). In the near generalization test trials, a red door and a yellow door appeared. A new object disappeared behind one of the doors. Participants were asked, “Which door would you open to find the [object]? Would you open the yellow door or the red door?” Participants chose their responses, either the door that the object disappeared behind (the *BC response*) or the other door (the *BI response*). In the far generalization test trials, an object moved horizontally and disappeared behind one of two screens; participants were asked to predict whether the object was behind the screen it went behind (*BC response*) or the other screen (*BI response*).

*Explanation questions*. At the end of the experiment, participants in the BC and BI conditions were shown one of the familiarization trials again, and were asked, “Why did that happen? Why did the [object] appear at that location?” Participants in the PB condition were shown one of the easy test trials again, and were asked, “You predicted that the [object] is behind [participants’ response]. Why is that the case? Why is the [object] behind [participants’ response]?” Participants typed in their answers.

#### Solidity principle

*Familiarization trials.* In each familiarization trial, a dark grey wall appeared and rotated 180 degrees to show that there was no hole in the wall. A green screen was placed in front of the wall and occluded the lower half of the wall. An object moved behind the screen (In Experiment 1, the object started moving on its own; In Experiments 2 and 3, the object went down a ramp). In the PB condition, the screen was not removed to reveal the location of the object. In the other two conditions, the screen was removed. The object was either on the side of the wall that it went behind (BC condition) or on the other side of the wall (BI condition). A different object was used in each trial.

*Test trials.* Participants completed the test trials next. In the easy test trials, a new object moved behind the green screen. A purple heart and an orange heart appeared in front of the screen, one on each side of the wall. Participants were asked, “Where do you think you will find the [object] now? Behind the purple heart or behind the orange heart?” Participants chose their responses, either the side of the wall that the object went behind (the *BC response*) or the other side of the wall (the *BI response*). In the near generalization test trials, two doors (side by side, with no gap in between) were placed in front of the wall and occluded the lower half of the wall. A new object moved behind the doors. Participants were asked, “Which door would you open to find the [object]? Would you open the yellow door or the red door?” Participants chose their responses, either the side of the wall that the object went behind (the *BC response*) or the other side of the wall (the *BI response*). In the far generalization test trials, a vertical wall and two horizontal walls appeared. Then, a screen covered the walls. An object disappeared behind the screen, and participants were asked to predict whether the object was above the first horizontal wall (*BC response*) or below the first horizontal wall (*BI response*).

*Explanation Questions*. At the end of the experiment, participants in the BC and BI conditions were shown one of the familiarization trials again, and were asked, “Why did that happen? Why did the [object] appear at that location?” Participants in the PB condition were shown one of the easy test trials again, and were asked, “You predicted that the [object] is behind [participants’ response]. Why is that the case? Why is the [object] behind [participants’ response]?” Participants typed in their answers.

#### Contact principle

We used different types of events in Experiment 1 and in Experiments 2 and 3. The events in Experiments 2 and 3 were more similar to those used in previous infant studies.

*Experiment 1: familiarization trials.* in each familiarization trial, participants were shown a blue box that can play music. An object was placed either on the toy (BC condition) or above the toy (BI condition), and immediately the toy lit up and played music for 5 s. A different object was used to activate the toy in each trial.

*Experiment 1: test trials.* Participants completed 2 easy test trials and 2 near generalization test trials next. In the easy test trials, a new object was placed next to the blue box. A red star and a yellow star appeared directly on top of the toy and above the toy. Participants were asked, “You can use the [object] to activate the toy and make it play music. Where would you put the [object]? Would you put it at the red star or the yellow star?” Participants chose their responses, either the location directly on top of the toy (the *BC response*) or the location above the toy (the *BI response*). In the near generalization test trials, a new, brown box and a new object appeared. Participants were asked, “The brown box is another music toy. You can use the [object] to activate the toy and make it play music. Where would you put the [object]? Would you put it at the red star or the yellow star?” Participants chose their responses, either the location directly on top of the toy (the *BC response*) or the location above the toy (the *BI response*).

*Experiments 2 and 3: familiarization trials.* In each familiarization trial, a yellow car moved toward an object and launched the object either by contacting it (BC condition) or at a distance (BI condition). In the PB condition, a screen blocked the view between the yellow car and the object so that participants could not see whether the yellow car contacted the other object or not. The yellow car launched a different object in each trial.

*Experiments 2 and 3: test trials.* Participants completed the test trials next. In the easy test trials, the yellow car and a new object appeared. A red star and a yellow star appeared right next to the object and at a short distance from it. Participants were asked, “The yellow toy car can launch the [object]. Where should the yellow toy car stop to launch the [object]? Should it stop at the yellow star or at the red star?” Participants chose their responses, either the location right next to the object (the *BC response*) or the location at a distance (the *BI response*). In the near generalization test trials, a new wheeled toy (e.g., a helicopter) and a new object appeared. A red star and a yellow star appeared right next to the object and at a short distance from it. Participants were asked, “The helicopter toy can launch the [object]. Where should the helicopter toy stop to launch the [object]? Should it stop at the yellow star or at the red star?” Participants chose their responses, either the location right next to the object (the *BC response*) or the location at a distance (the *BI response*). In the far generalization test trials, participants were shown a music toy and were told that it can be activated by objects; they chose whether they would activate the toy by placing an object directly on top of the toy (*BC response*) or hovering above the toy (*BI response*).

*Experiments 2 and 3: explanation questions*. At the end of the experiment, participants in the BC and BI conditions were shown one of the familiarization trials again, and were asked, “Why did that happen? Why did the yellow car launch the [object]?” Participants in the PB condition were shown one of the easy test trials again, and were asked, “You predicted that the yellow car should stop at [participants’ response] to make the [object] go. Why is that the case? Why would the yellow car make the [object] go if it stops [participants’ response]?” Participants typed in their answers.

### Experiments 4–6

#### Participants

In Experiment 4, we tested 24 children between the ages of 4 and 6 years (mean age = 5.04; range = 4.08 to 6.92; SD = 0.82; 11 females). In Experiment 5, we tested 36 children between the ages of 4 and 6 years (mean age = 4.85; range = 4 to 6.83; SD = 0.80; 18 females, 15 males, and 3 of unknown gender). In Experiment 6, we tested 36 children between the ages of 4 and 6 years (mean age = 5.41; range = 4 to 6.92; SD = 0.88; 14 females and 22 males).

Participants were tested via Zoom. Parents of the participants provided written informed consent prior to the experimental session. Children received an electronic certificate after they completed the experiments.

#### Design and procedure

The design and procedure of Experiments 4–6 were the same as Experiments 1–3, respectively. An experimenter showed the stimuli videos to children via Zoom, children told the experimenter their responses verbally, and the experimenter clicked on the answers. Children’s answers for the explanation questions were transcribed from the video recordings.

### Experiments 7–9

#### Participants

In Experiment 7, we tested 47 adults (mean age = 30 years; range = 18 to 55; *SD* = 9.2; 25 females) on Prolific. In Experiment 8, we tested 60 adults (mean age = 33 years; range = 18 to 54; *SD* = 9.41; 35 females) on Prolific. In Experiment 9, we tested 82 undergraduate students who were enrolled in psychology courses (mean age = 20.28 years; range = 18 to 36; *SD* = 2.54; 65 females, 15 males, 2 of unknown gender). Participants were tested and compensated in the same manner as in Experiments 1–3.

#### Design

In all 3 experiments, participants were tested on 3 principles (Efficiency, Goal, and Sampling) in counterbalanced orders. The designs were the same as Experiments 1 to 3 (i.e., the design of Experiment 7 is the same as Experiment 1; the design of Experiment 8 is the same as Experiment 2; the design of Experiment 9 is the same as Experiment 3). The only difference is that participants observed 3 familiarization trials (instead of 4) in Experiment 7.

#### Stimuli and procedure

At the beginning of the experiment, participants were shown the following instructions: “In the following pages, you will watch some videos and observe how agents behave. Then, you will be asked to make some predictions.”

##### Efficiency principle

*Familiarization trials.* In each familiarization trial, a grey wall and 2 agents (i.e., geometric shapes with eyes) appeared. One agent moved toward the other agent by jumping over the wall. Then, the wall was moved to the side. In the PB condition, the agent did not go towards the other agent. In the other two conditions, the agent moved toward the other agent by taking a straight path (BC condition) or a jumping path (BI condition). The goal was a different agent in each familiarization trial.

*Test trials.* Participants completed the test trials next. In the easy test trials, the same agent moved toward a new geometric-shaped agent by jumping over a wall. Then, the wall was moved to the side. A red path and a blue path indicated the straight path (the *BC response*) and the jumping path (the *BI response*). Participants were asked, “[The agent] wants to play with [the other agent]. Which path do you think [the agent] will take to get to [the other agent]? The blue path or the red path?” Participants chose their responses, either the blue, straight path (the *BC response*) or the red, jumping path (the *BI response*). In the near generalization test trials, a new geometric-shaped agent moved toward another geometric-shaped agent by jumping over a wall. Then, the wall was moved to the side. Participants chose which path the new agent would take to get to the other agent, either the blue, straight path (the *BC response*) or the red, jumping path (the *BI response*). In the far generalization test trials, an animal moved toward another animal by jumping over a wall. Then, the wall was moved to the side. Participants chose which path the animal would take to get to the animal, either the blue, straight path (the *BC response*) or the red, jumping path (the *BI response*).

*Explanation questions*. At the end of the experiment, participants in the BC and BI conditions were shown one of the familiarization trials again, and were asked, “Why did that happen? When the wall is moved to the side, why did [the agent] take the jumping path/the straight path to get to [the other agent]?” Participants in the PB condition were shown one of the easy test trials again, and were asked, “You predicted [the agent] would take the red path/the blue path to get to [the other agent] when there is no wall. Why is that the case? Why would [the agent] take the red path/the blue path?” Participants typed in their answers.

##### Goal principle

*Familiarization trials.* In each familiarization trial, an agent and 2 objects appeared. The agent moved toward one of two objects and took the object with her 3 times. Then, the two objects switched locations. In the PB condition, the agent did not go toward either object. In the other two conditions, the agent took the old object at the new location (BC condition) or the new object at the old location (BI condition). A different pair of objects was used in each familiarization trial.

*Test trials.* Participants completed the test trials next. In the easy test trials, a new pair of objects appeared. The same agent took one of the objects 3 times. Then the two objects switched locations. Participants were asked, “Which toy do you think [the agent] will take? The [object 1] or the [object 2]?” Participants chose their responses, either the old object at the new location (*BC response*) or the new object at the old location (*BI response*). In the near generalization test trials, a new geometric-shaped agent and a new pair of objects appeared. The agent took one of the objects 3 times. Then the two objects switched locations. Participants chose which toy the agent will take, either the old object at the new location (*BC response*) or the new object at the old location (*BI response*). In the far generalization test trials, an animal and a new pair of objects appeared. The animal took one of the objects 3 times. Then the two objects switched locations. Participants chose which toy the animal will take, either the old object at the new location (*BC response*) or the new object at the old location (*BI response*).

*Explanation questions*. At the end of the experiment, participants in the BC and BI conditions were shown one of the familiarization trials again, and were asked, “Why did that happen? During the last time, why did [the agent] take the [object 1/object 2]?” Participants in the PB condition were shown one of the easy test trials again, and were asked, “You predicted that [the agent] would take [object 1/object 2] when the toys switched places. Why is that the case? Why would [the agent] take [object 1/object 2]?” Participants typed in their answers.

##### Sampling principle.

*Familiarization trials.* In each familiarization trial, an agent and a box of objects appeared. The box contained 7 objects of one type (minority type) and 31 objects of the other type (majority type). The agent picked out 4 objects of the minority type from the box and put them into a small box in front of the agent. Then an object of the minority type and an object of the majority type appeared, equidistant from the agent. In the PB condition, the agent did not go towards either object. In the other two conditions, the agent moved toward the minority type (BC condition) or the majority type (BI condition). A different toy box was used in each familiarization trial.

*Test trials.* Participants completed the test trials next. In the easy test trials, the same agent sampled 4 objects of the minority type from a new toy box. Then an object of the minority type and an object of the majority type appeared, equidistant from the agent. Participants were asked, “Which toy do you think [the agent] likes better, [object 1] or [object 2]?” Participants chose their responses, either the minority-type object (*BC response*) or the majority-type object (*BI response*). In the near generalization test trials, a new geometric-shaped agent sampled 4 objects of the minority type from a new toy box. Then an object of the minority type and an object of the majority type appeared, equidistant from the agent. Participants chose which toy the agent liked better, either the minority-type object (*BC response*) or the majority-type object (*BI response*). In the near generalization test trials, an animal sampled 4 objects of the minority type from a new toy box. Then an object of the minority type and an object of the majority type appeared, equidistant from the agent. Participants chose which toy the animal liked better, either the minority-type object (*BC response*) or the majority-type object (*BI response*).

*Explanation questions*. At the end of the experiment, participants in the BC and BI conditions were shown one of the familiarization trials again, and were asked, “Why did that happen? Why did [the agent] go toward [object 1/object 2]?” Participants in the PB condition were shown one of the easy test trials again, and were asked, “You predicted that [the agent] likes [object 1/object 2] better. Why is that the case? Why does [the agent] like [object 1/object 2]?” Participants typed in their answers.

### Experiments 10–12

#### Participants

In Experiment 10, we tested 24 children between the ages of 4 and 6 years (mean age = 5.04; range = 4.08 to 6.92; SD = 0.82; 11 females). In Experiment 11, we tested 36 children between the ages of 4 and 6 years (mean age = 5.12; range = 4 to 6.75; SD = 0.92; 11 females, 23 males, and 2 of unknown gender). In Experiment 12, we tested 36 children between the ages of 4 and 6 years (mean age = 4.99; range = 4 to 6.83; SD = 0.87; 22 females and 14 males). Participants were tested and compensated in the same manner as in Experiments 4–6.

#### Design and procedure

The design and procedure of Experiments 10–12 were the same as Experiments 7–9, respectively.

## Supplementary Information

Below is the link to the electronic supplementary material.

Supplementary Material 1

## Data Availability

The datasets generated during the current study are available in the Open Science Framework (OSF) repository, [https://osf.io/f2dk3/overview?view_only=38afbc85d0a34e25ab86cfb28e4edbe8] .
