# Supplementary material for: Children and adults can suspend core principles about objects and agents given a small amount of counterevidence on screen
Source: Sci Rep. 2025 Dec 13;16:1834. doi: 10.1038/s41598-025-31534-9 (PMC12804722; doi:10.1038/s41598-025-31534-9)
Supplement: Supplementary file 1 — Supplementary Material 1 [file 41598_2025_31534_MOESM1_ESM.docx]

Supplementary Information for

Children and Adults Can Suspend Core Principles About Objects and Agents Given a Small Amount of Counterevidence on Screen

Supporting Results

Experiments 1—3 (Physical principles: Adults)

We used mixed-effect logistic regression to predict participants’ binary choice (BI = 1, BC = 0) from condition, principle, test trial type, experiment, and their interactions, while controlling for the random effects of individual participants. The best-fitting model included the interaction of condition and principle, and the interaction of condition and test trial type as predictors.

Most importantly, for all 3 principles, participants were more likely to choose the *BI response* in the BI condition than in the Baseline condition (Continuity: *β* = 8.91, *SE* = 0.79, *p* < .001; Solidity: *β* = 7.00, *SE* = 0.66, *p* < .001; Contact: *β* = 4.98, *SE* = 0.63, *p* < .001) and the BC condition (Continuity: *β* = 8.57, *SE* = 0.67, *p* < .001; Solidity: *β* = 8.21, *SE* = 0.65, *p* < .001; Contact: *β* = 6.20, *SE* = 0.62, *p* < .001). For the Continuity and the Solidity principles, participants’ responses did not differ between the BC condition and the Baseline condition (Continuity: *β* = 0.42, *SE* = 0.79, *p* = .59; Solidity: *β* = -1.20, *SE* = 0.64, *p* = .062). For the Contact principle, they were less likely to choose the *BI response* in the BC condition than in the Baseline condition (*β* = -1.25, *SE* = 0.63, *p* = .046).

The interaction between condition and principle showed that, in the Baseline condition, compared to the Continuity principle, participants were more likely to choose the *BI response* for the Solidity (*β* = 1.98, *SE* = 0.57, *p* < .001) and the Contact principle (*β* = 2.40, *SE* = 0.56, *p* < .001). In the BC condition, participants were more likely to choose the *BI response* for the Contact principle than for the Continuity principle (*β* = 0.87, *SE* = 0.35, *p* = .012). In the BI condition, participants were less likely to choose the *BI response* for the Contact principle compared to the Continuity principle (*β* = -1.59, *SE* = 0.23, *p* < .001) and the Solidity principle (*β* = -1.63, *SE* = 0.23, *p* < .001).

The interaction between condition and test trial type showed that, while participants’ performance did not differ across test trial types in the Baseline condition, in the BC condition, they were more likely to choose the *BI response* in the far generalization test trials than in the test trials (*β* = 0.88, *SE* = 0.40, *p* = .028); and in the BI condition, they were less likely to choose the *BI response* in the near generalization test trials than in the test trials (*β* = -0.44, *SE* = 0.21, *p* = .038), and less likely to choose the *BI response* in the far generalization test trials than in the test trials (*β* = -1.47, *SE* = 0.28, *p* < .001) and the near generalization test trials (*β* = -1.03, *SE* = 0.27, *p* < .001).

There was no significant effect of experiment, suggesting that the different stimuli sets did not affect participants’ choices in the test trials.

To examine the effect of the amount of evidence on participants’ choices, we analyzed the BC condition data across the 3 experiments and the BI condition data across the 3 experiments, respectively. The amount of evidence did not affect adults’ *BI response* in the BC condition or the BI condition.

Experiments 4—6 (Physical principles: Children)

We used mixed-effect logistic regression to predict participants’ binary choice (BI = 1, BC = 0) from condition, principle, test trial type, experiment, and their interactions, while controlling for the random effects of individual participants. The best-fitting model included the interaction of condition and principle as predictors.

Most importantly, for all 3 principles, participants were more likely to choose the *BI response* in the BI condition than in the Baseline condition (Continuity: *β* = 5.00, *SE* = 0.59, *p* < .001; Solidity: *β* = 1.29, *SE* = 0.39, *p* < .001; Contact: *β* = 1.66, *SE* = 0.40, *p* < .001) and the BC condition (Continuity: *β* = 4.72, *SE* = 0.48, *p* < .001; Solidity: *β* = 3.04, *SE* = 0.39, *p* < .001; Contact: *β* = 1.93, *SE* = 0.37, *p* < .001). For the Continuity and the Contact principles, participants’ responses did not differ between the BC condition and the Baseline condition (Continuity: *β* = 0.28, *SE* = 0.64, *p* = .66; Contact: *β* = -0.28, *SE* = 0.41, *p* = .50). For the Solidity principle, participants were less likely to choose the *BI response* in the BC condition than in the Baseline condition (*β* = -1.76, *SE* = 0.41, *p* < .001).

The interaction between condition and principle showed that, in the Baseline and the BC conditions, compared to the Continuity principle, participants were more likely to choose the *BI response* for the Solidity (Baseline condition: *β* = 3.35, *SE* = 0.51, *p* < .001; BC condition: *β* = 1.32, *SE* = 0.39, *p* < .001) and the Contact principle (Baseline condition: *β* = 2.23, *SE* = 0.52, *p* < .001; BC condition: *β* = 1.67, *SE* = 0.39, *p* < .001). In the BI condition, participants were less likely to choose the *BI response* for the Contact principle compared to the Continuity principle (*β* = -1.12, *SE* = 0.26, *p* < .001) and the Solidity principle (*β* = -0.76, *SE* = 0.25, *p* = .002).

There was no significant effect of test trial type (test trials vs. near generalization test trials vs. far generalization test trials), suggesting that children generalized the new beliefs to new objects and events.

There was no significant effect of experiment, suggesting that the different stimuli sets did not affect participants’ choices in the test trials.

To examine the effect of the amount of evidence on participants’ choices, we analyzed the BC condition data across the 3 experiments and the BI condition data across the 3 experiments, respectively. The amount of evidence did not affect children’s *BI response* in the BC condition or the BI condition.

Experiments 1—6 (Physical principles: Age group comparison)

We used mixed-effect logistic regression to predict participants’ binary choice (BI = 1, BC = 0) from condition, principle, test trial type, age group (adults vs. children), and their interactions, while controlling for the random effects of individual participants. The best-fitting model included the three-way interaction of condition, principle, and age group, and the two-way interaction of condition and test trial type as predictors.

Most importantly, for all 3 principles, participants were more likely to choose the *BI response* in the BI condition than in the Baseline condition (Continuity: *β* = 8.08, *SE* = 0.68, *p* < .001; Solidity: *β* = 6.14, *SE* = 0.51, *p* < .001; Contact: *β* = 4.30, *SE* = 0.48, *p* < .001) and the BC condition (Continuity: *β* = 7.36, *SE* = 0.52, *p* < .001; Solidity: *β* = 6.87, *SE* = 0.49, *p* < .001; Contact: *β* = 5.10, *SE* = 0.46, *p* < .001); their choices did not differ between the Baseline and the BC conditions (Continuity: *β* = -0.98, *SE* = 0.53, *p* = .065, Solidity: *β* = -0.98, *SE* = 0.53, *p* = .065; Contact: *β* = -0.82, *SE* = 0.50, *p* = .10).

The three-way interaction of condition, principle, and age group (adult vs. child) showed that, in the Baseline and BC conditions, children were more likely than adults to choose the *BI response* for the Solidity principle (Baseline: *β* = 3.16, *SE* = 0.56, *p* < .001; BC: *β* = 1.64, *SE* = 0.51, *p* = .001) and the Contact principle (Baseline: *β* = 1.57, *SE* = 0.56, *p* = .005; BC: *β* = 1.69, *SE* = 0.49, *p* < .001). In the BI condition, children were less likely than adults to choose the *BI response* for the Continuity principle (*β* = -1.01, *SE* = 0.45, *p* = .024) and the Solidity principle (*β* = -1.32, *SE* = 0.44, *p* = .003).

The interaction of condition and test trial type showed that, in the Baseline condition, participants were less likely to choose the *BI response* in the far generalization test trials than in the test trials (*β* = -0.76, *SE* = 0.34, *p* = .024) and the near generalization test trials (*β* = -0.67, *SE* = 0.33, *p* = .044). In the BC condition, participants were more likely to choose the *BI response* in the far generalization test trials than in the test trials (*β* = 0.63, *SE* = 0.29, *p* = .029). In the BI condition, participants were less likely to choose the *BI response* in the far generalization test trials than in the test trials (*β* = -0.95, *SE* = 0.21, *p* < .001) and the near generalization test trials (*β* = -0.78, *SE* = 0.21, *p* < .001).

To examine the effect of the amount of evidence on participants’ choices, we analyzed the BC condition data across the 6 experiments and the BI condition data across the 6 experiments, respectively. The amount of evidence did not affect participants’ *BI response* in the BC condition or the BI condition.

Experiment 1—6 (Physical principles): Explanation data

At the end of the experiment, participants in the BI condition were asked to explain one of the belief-violating events they observed for each principle. We coded participants’ explanations into 4 categories: *accept evidence*, *explain away*, *pattern*, or *other*. An explanation was coded as *accept evidence* if the explanation showed that the participant had accepted the counterevidence to the target principle in the counterevidence (e.g., “the car can go through the wall” for Solidity). An explanation was coded as *explain away* if the participant explained the counterevidence with reasons that would not involve any inconsistency with the target principle (e.g., “the first wall was further away towards the back” for Solidity). An explanation was coded as *pattern* if the explanation simply noted the pattern in the evidence without mentioning the inconsistency with the relevant principle or explicitly accepting the counterevidence (e.g., “it has been the pattern the whole time”). Lastly, explanations that cannot be categorized into the first 3 categories were coded as *other* (e.g., “it is just the way it goes”, “I don’t know”).

The distribution of different types of explanations did not differ between adults and children (*p* > .10).

For adults, those who provided *accept evidence* and *pattern* explanations were more likely to choose the *BI response* for the principle compared to participants who provided *explain away* explanations (*accept evidence*: *β* = 0.98, *SE* = 0.45, *p* = .03; *pattern*: *β* = 1.52, *SE* = 0.63, *p* = .02) or *other* explanations (*accept evidence*: *β* = 0.96, *SE* = 0.47, *p* = .04; *pattern*: *β* = 1.50, *SE* = 0.67, *p* = .03) for that principle. For children, with fewer explanations provided, the type of explanation did not significantly predict their choices in the test trials.

We next analyzed the combined explanation data for adults and children. Participants who provided *pattern* explanations were more likely to choose the *BI response* for the principle compared to participants who provided *explain away* explanations (*β* = 1.55, *SE* = 0.59, *p* = .008) or *other* explanations (*β* = 1.69, *SE* = 0.62, *p* = .01). Participants who provided *accept evidence* explanations were marginally more likely to choose the *BI response* for the principle compared to participants who provided *explain away* (*β* = 0.40, *SE* = 0.23, *p* = .09) or *other* explanations (*β* = 0.54, *SE* = 0.29, *p* = .07) for that principle. When we grouped *accept evidence* or *pattern* explanations into a single category, and grouped *explain away* and *other* explanations into a single category, we found that participants who provided *accept evidence* or *pattern* explanations were more likely to choose the *BI response* for the principle compared to participants who provided *explain away* or *other* explanations (*β* = 0.57, *SE* = 0.21, *p* = .006).

Experiments 7—9 (Psychological principles: Adults)

We used mixed-effect logistic regression to predict participants’ binary choice (BI = 1, BC = 0) from condition, principle, trial type, experiment, and their interactions, while controlling for the random effects of individual participants. The best-fitting model included condition, and the interaction of principle and experiment as predictors.

Most importantly, for all 3 principles participants were more likely to choose the *BI response* in the BI condition than in the Baseline (*β* = 4.78, *SE* = 0.53, *p* < .001) and the BC conditions (*β* = 6.00, *SE* = 0.52, *p* < .001); they were less likely to choose the *BI response* in the BC condition than in the Baseline condition (*β* = -1.22, *SE* = 0.52, *p* = .02).

The interaction of principle and experiment showed that, in Experiment 7, participants were more likely to choose the *BI response* for the Efficiency principle than for the Sampling principle (*β* = 1.37, *SE* = 0.34, *p* < .001). In Experiments 8 and 9, participants were more likely to choose the *BI response* for the Efficiency principle (Experiment 8: *β* = 3.48, *SE* = 0.41, *p* < .001; Experiment 9: *β* = 1.97, *SE* = 0.24, *p* < .001) and for the Goal principle (Experiment 8: *β* = 2.31, *SE* = 0.38, *p* < .001; Experiment 9: *β* = 1.46, *SE* = 0.23, *p* < .001), compared to the Sampling principle.

There was no significant effect of test trial type (test trials vs. near generalization test trials vs. far generalization test trials), suggesting that adults generalized the new beliefs to new agents. There was no significant effect of experiment, suggesting that the different stimuli sets did not affect participants’ choices in the test trials.

To examine the effect of the amount of evidence on participants’ choices, we analyzed the BC condition data across the 3 experiments and the BI condition data across the 3 experiments, respectively.

We used mixed-effect logistic regression to predict participants’ binary choice (BI = 1, BC = 0) in the BC condition from the amount of evidence (3 pieces of evidence in Experiment 7; 6 pieces of evidence in Experiment 8, and 9), while controlling for the random effects of individual participants. The amount of evidence did not affect participants’ *BI response* in the BC condition.

We used mixed-effect logistic regression to predict participants’ binary choice (BI = 1, BC = 0) in the BI condition from the amount of evidence (3 pieces of evidence in Experiment 7; 6 pieces of evidence in Experiment 8, and 9), while controlling for the random effects of individual participants. We found a significant effect of the amount of evidence. In the BI condition, when participants were given 6 pieces of counterevidence, they were more likely to choose the *BI response* in the test trials, compared to when they were given 3 pieces of counterevidence (*β* = 0.83, *SE* = 0.42, *p* = .046).

Experiments 10—12 (Psychological principles: Children)

We used mixed-effect logistic regression to predict participants’ binary choice (BI = 1, BC = 0) from condition, principle, trial type, experiment, and their interactions, while controlling for the random effects of individual participants. The best-fitting model included the three-way interaction of condition, principle, and experiment as predictors.

Most importantly, for the Goal and the Sampling principles, children were more likely to choose the *BI response* in the BI condition than in the Baseline (Goal: *β* = 3.57, *SE* = 0.90, *p* < .001; Sampling: *β* = 1.53, *SE* = 0.58, *p* = .009;) and the BC (Goal: *β* = 4.71, *SE* = 0.90, *p* < .001; Sampling: *β* = 1.59, *SE* = 0.59, *p* = .007) conditions, and their responses did not differ between the Baseline and the BC conditions (Goal: *β* = -1.13, *SE* = 0.58, *p* = .053; Sampling: *β* = -.05, *SE* = 0.59, *p* = 0.93). For the Efficiency principle, children were marginally more likely to choose the *BI response* in the BI condition than in the Baseline condition (*β* = 1.28, *SE* = 0.66, *p* = .053); their responses did not differ between the BC and the BI conditions (*β* = 0.40, *SE* = 0.64, *p* = .53), or between the Baseline and the BC conditions (*β* = 0.90, *SE* = 0.66, *p* = .17).

The three-way interaction of condition, principle, and experiment showed that, in the Baseline condition, children were more likely to choose the *BI response* for the Goal and the Efficiency principles than for the Sampling principle in Experiment 2 (Goal: *β* = 1.88, *SE* = 0.72, *p* = .009; Efficiency: *β* = 2.14, *SE* = 0.72, *p* = .003) and Experiment 3 (Goal: *β* = 1.16, *SE* = 0.38, *p* = .002; Efficiency: *β* = 0.95, *SE* = 0.37, *p* = .01). In the BC condition in Experiment 1, children were more likely to choose the *BI response* for Efficiency principle than for the other 2 principles (Goal: *β* = 1.44, *SE* = 0.47, *p* = .002; Sampling: *β* = 2.47, *SE* = 0.53, *p* < .001), and they were more likely to choose the *BI response* for Goal principle than for the Sampling principle (*β* = 1.03, *SE* = 0.52, *p* = .048); in the BC condition in Experiment 2, children were more likely to choose the *BI response* for Efficiency principle than for the other 2 principles (Goal: *β* = 2.70, *SE* = 0.65, *p* < .001; Sampling: *β* = 3.03, *SE* = 0.72, *p* < .001). In the BI condition in Experiment 1, children were more likely to choose the *BI response* for the Goal principles than for the Efficiency principle (*β* = 1.48, *SE* = 0.50, *p* = .003); in the BI condition in Experiment 3, children were more likely to choose the *BI response* for the Goal principles than for the other 2 principles (Efficiency: *β* = 2.98, *SE* = 0.79, *p* < .001; Sampling: *β* = 3.22, *SE* = 0.79, *p* < .001).

There was no significant effect of test trial type (test trials vs. near generalization test trials vs. far generalization test trials), suggesting that children generalized the new beliefs to new agents. There was no significant effect of experiment, suggesting that the different stimuli sets did not affect participants’ choices in the test trials.

To examine the effect of the amount of evidence on participants’ choices, we analyzed the BC condition data across the 3 experiments and the BI condition data across the 3 experiments, respectively. The amount of evidence did not affect children’s *BI response* in the BC condition or the BI condition.

Experiments 7—12 (Psychological principles: Age group comparison)

We used mixed-effect logistic regression to predict participants’ binary choice (BI = 1, BC = 0) from condition, principle, test trial type, age group (adults vs. children), and their interactions, while controlling for the random effects of individual participants. The best-fitting model included the three-way interaction of condition, principle, and age group as predictors.

Most importantly, adults were more likely to choose the *BI response* in the BI condition than in the Baseline (Efficiency: *β* = 4.18, *SE* = 0.43, *p* < .001; Goal: *β* = 3.68, *SE* = 0.42, *p* < .001; Sampling: *β* = 4.36 *SE* = 0.49, *p* < .001) and the BC conditions (Efficiency: *β* = 5.27, *SE* = 0.43, *p* < .001; Goal: *β* = 5.30, *SE* = 0.46, *p* < .001; Sampling: *β* = 5.03, *SE* = 0.52, *p* < .001); they were less likely choose the *BI response* in the BC condition than in the Baseline condition (Efficiency: *β* = -1.21, *SE* = 0.44, *p* = .006; Goal: *β* = -1.61, *SE* = 0.47, *p* < .001; not significant for Sampling: *β* = -0.71, *SE* = 0.60, *p* = .24).

Most importantly, children were also more likely to choose the *BI response* in the BI condition than in the Baseline (Efficiency: *β* = 1.25, *SE* = 0.53, *p* = .019; Goal: *β* = 2.80, *SE* = 0.56, *p* < .001; Sampling: *β* = 2.66, *SE* = 0.55, *p* < .001) and the BC (Efficiency: *β* = 0.96, *SE* = 0.47, *p* = .040; Goal: *β* = 3.63, *SE* = 0.51, *p* < .001; Sampling: *β* = 2.65, *SE* = 0.49, *p* < .001) conditions, and their responses did not differ between the Baseline and the BC conditions (Efficiency: *β* = 0.31, *SE* = 0.53, *p* = .56; Goal: *β* = -0.85, *SE* = 0.54, *p* = .12; Sampling: *β* = -.001, *SE* = 0.56, *p* = 1.00).

In the Baseline and the BC conditions, children were more likely than adults to choose the *BI response* for all 3 principles (Baseline: Efficiency: *β* = 1.41, *SE* = 0.52, *p* = .006; Goal: *β* = 1.82, *SE* = 0.52, *p* = .005; Sampling: *β* = 2.36, *SE* = 0.60, *p* < .001; BC: Efficiency: *β* = 2.83, *SE* = 0.46, *p* < .001; Goal: *β* = 2.55, *SE* = 0.50, *p* < .001; Sampling: *β* = 3.12, *SE* = 0.57, *p* < .001). In the BI condition, children were less likely than adults to choose the *BI response* for the Efficiency principle (*β* = -1.51, *SE* = 0.43, *p* < .001), and more likely than adults to choose the *BI response* for the Goal principle (*β* = 0.95, *SE* = 0.46, *p* = .037).

To examine the effect of the amount of evidence on participants’ choices, we analyzed the BC condition data across the 6 experiments and the BI condition data across the 6 experiments, respectively. The amount of evidence did not affect participants’ *BI response* in the BC condition or the BI condition.

Experiment 7—12 (Psychological principles): Explanation data

We coded participants’ explanations into 4 categories, *accept evidence*, *explain away*, *pattern*, or *other*, based on the same criteria as with the physical principles.

The distribution of explanation types differed for adults and children. Adults were more likely to provide *accept evidence* than *explain away* explanations compared to children (*β* = 1.14, *SE* = 0.44, *p* = .01).

For adults, they were more likely to choose the *BI response* if they provided *accept evidence* explanations for that principle, compared to if they provided any other types of explanations (*explain away*: *β* = 1.44, *SE* = 0.35, *p* < .001; *pattern*: *β* = 1.05, *SE* = 0.44, *p* = .017; *other*: *β* = 1.96, *SE* = 0.34, *p* < .001).

For children, those who provided *accept evidence* explanations were more likely to choose the *BI response* for the principle compared to those who provided *other* explanations (*β* = 0.95, *SE* = 0.34, *p* = .01). Children’s data were noisier than that of adults: those who provided *explain away* explanations were more likely to choose the *BI response* for the principle compared to those who provided *accept evidence* explanations (*β* = 0.89, *SE* = 0.43, *p* = .04) and *other* explanations (*β* = 1.84, *SE* = 0.43, *p* < .001).

We next analyzed the combined explanation data of adults and children. Participants were more likely to choose the *BI response* if they provided *accept evidence* explanations for that principle, compared to if they provided *explain away* (*β* = 0.64, *SE* = 0.25, *p* = .01), or *other* explanations (*β* = 1.62, *SE* = 0.24, *p* < .001).

Experiment 1—12: Comparison of explanation data across domains

We compared the distribution of explanation types across the two domains. Participants were more likely to provide *accept evidence* explanations for the psychological principles than *explain away* explanations (*β* = 1.48, *SE* = 0.28, *p* < .001) and *pattern* explanations (marginally significant: *β* = 0.79, *SE* = 0.41, *p* = .052), compared to the physical principles; and they were less likely to provide *explain away* explanations for the psychological principles than *other* explanations, compared to the physical principles (*β* = -0.95, *SE* = 0.34, *p* = .005).
